# Optimizing *in silico* drug discovery: simulation of connected differential expression signatures and applications to benchmarking

**DOI:** 10.1093/bib/bbae299

**Published:** 2024-06-27

**Authors:** Catalina Gonzalez Gomez, Manuel Rosa-Calatrava, Julien Fouret

**Affiliations:** CIRI, Centre International de Recherche en Infectiologie, Team VirPath, Univ Lyon, 14 Inserm, U1111, Université Claude Bernard Lyon 1, CNRS, UMR5308, ENS de Lyon, F-15 69007 Lyon, Rhône-Alpes, France; International Associated Laboratory RespiVir France—Canada, Centre de Recherche en Infectiologie, Faculté de Médecine RTH Laennec 69008 Lyon, Université Claude Bernard Lyon 1, Université de Lyon, INSERM, CNRS, ENS de Lyon, France, Centre Hospitalier Universitaire de Québec - Université Laval, QC G1V 4G2 Québec, Canada; Nexomis, Faculté de Médecine RTH Laennec, Université Claude Bernard Lyon 1, Université de Lyon, 7 Rue Guillaume Paradin, 69008 Lyon, Rhône-Alpes, France; Signia Therapeutics, 60 Avenue Rockefeller, 69008 Lyon, Rhône-Alpes, France; CIRI, Centre International de Recherche en Infectiologie, Team VirPath, Univ Lyon, 14 Inserm, U1111, Université Claude Bernard Lyon 1, CNRS, UMR5308, ENS de Lyon, F-15 69007 Lyon, Rhône-Alpes, France; International Associated Laboratory RespiVir France—Canada, Centre de Recherche en Infectiologie, Faculté de Médecine RTH Laennec 69008 Lyon, Université Claude Bernard Lyon 1, Université de Lyon, INSERM, CNRS, ENS de Lyon, France, Centre Hospitalier Universitaire de Québec - Université Laval, QC G1V 4G2 Québec, Canada; Nexomis, Faculté de Médecine RTH Laennec, Université Claude Bernard Lyon 1, Université de Lyon, 7 Rue Guillaume Paradin, 69008 Lyon, Rhône-Alpes, France; VirNext, Faculté de Médecine RTH Laennec, Université Claude Bernard Lyon 1, Université de Lyon, 7 Rue Guillaume Paradin, 69008 Lyon, Rhône-Alpes, France; CIRI, Centre International de Recherche en Infectiologie, Team VirPath, Univ Lyon, 14 Inserm, U1111, Université Claude Bernard Lyon 1, CNRS, UMR5308, ENS de Lyon, F-15 69007 Lyon, Rhône-Alpes, France; International Associated Laboratory RespiVir France—Canada, Centre de Recherche en Infectiologie, Faculté de Médecine RTH Laennec 69008 Lyon, Université Claude Bernard Lyon 1, Université de Lyon, INSERM, CNRS, ENS de Lyon, France, Centre Hospitalier Universitaire de Québec - Université Laval, QC G1V 4G2 Québec, Canada; Nexomis, Faculté de Médecine RTH Laennec, Université Claude Bernard Lyon 1, Université de Lyon, 7 Rue Guillaume Paradin, 69008 Lyon, Rhône-Alpes, France; Signia Therapeutics, 60 Avenue Rockefeller, 69008 Lyon, Rhône-Alpes, France

**Keywords:** connectivity score, drug repurposing, differential expression signature, simulation tool, benchmarking

## Abstract

**Background:**

We present a novel simulation method for generating connected differential expression signatures. Traditional methods have struggled with the lack of reliable benchmarking data and biases in drug–disease pair labeling, limiting the rigorous benchmarking of connectivity-based approaches.

**Objective:**

Our aim is to develop a simulation method based on a statistical framework that allows for adjustable levels of parametrization, especially the connectivity, to generate a pair of interconnected differential signatures. This could help to address the issue of benchmarking data availability for connectivity-based drug repurposing approaches.

**Methods:**

We first detailed the simulation process and how it reflected real biological variability and the interconnectedness of gene expression signatures. Then, we generated several datasets to enable the evaluation of different existing algorithms that compare differential expression signatures, providing insights into their performance and limitations.

**Results:**

Our findings demonstrate the ability of our simulation to produce realistic data, as evidenced by correlation analyses and the log_2_ fold-change distribution of deregulated genes. Benchmarking reveals that methods like extreme cosine similarity and Pearson correlation outperform others in identifying connected signatures.

**Conclusion:**

Overall, our method provides a reliable tool for simulating differential expression signatures. The data simulated by our tool encompass a wide spectrum of possibilities to challenge and evaluate existing methods to estimate connectivity scores. This may represent a critical gap in connectivity-based drug repurposing research because reliable benchmarking data are essential for assessing and advancing in the development of new algorithms. The simulation tool is available as a R package (General Public License (GPL) license) at https://github.com/cgonzalez-gomez/cosimu.

## Introduction

The field of pharmaceutical research has long relied on the use of differential whole-transcriptomic signatures to better understand the underlying mechanism of an external stimulus such as a pathogenic determinant (ex: virus infection) or the treatment by a small molecule. These signatures, which represent the global measurement of expression values for multiple tags, are often compared using a variety of methods, including the concept of connectivity. The Connectivity Map (CMAP) database [1] and its scaled-up version part of the National Institutes of Health (NIH) Library of Integrated Network-Based Cellular Signatures (LINCS) Consortium [2], holding the signatures for thousands of small molecules, has been extensively used either to predict the mode of action of new drugs or to perform drug repurposing [3].

Datasets with known drug–drug similarities have been used to evaluate different signature matching algorithms based on CMAP [4, 5] or LINCS data [6]. One can consider pairs of molecules with similar functions as a standard for benchmarking the estimation of positive connectivity [7]. However, in the context of using connectivity estimation for drug repurposing, there are no clear standard to benchmark different methods—on top of that—there are contradictory approaches on how drug and disease signature should be compared together [8].

To overcome these limitations, several evaluation datasets have been proposed with pairs of drug–disease signatures based on known indications [9]. We have identified two potential biases in the way the drug–disease pair are labeled. First, small molecules that have not been reported nor used as therapeutics for a disease are reported as negative in place of unknown. Second, although whole-transcriptome expression signature is more prompt to reflect a global effect of small molecule, drug discovery has long relied on single disease-specific target rather than the concept of polypharmacology in which a drug can influence multiple targets rather than only one [10]. In other words, many drug–disease pairs labeled as positive are not predictable using a signature-based method. Moreover, the single or multiple target–based drug discovery using molecular docking was still a trend in the last decades [11] and the drug-induced gene expression signature is not likely to predict its therapeutic effect. Recently, a dataset has been proposed to overcome this labeling bias by leveraging data from a preclinical efficacy screening assay [12]. Nevertheless, it may not be extrapolated to other diseases.

Overall, the lack of reliable benchmarking data makes it difficult to evaluate rigorously the effectiveness of the connectivity-based methods. To address this issue, we have developed a method for the simulation of connected differential expression signatures.

In this paper, we present the mathematical model and the algorithm to generate interconnected signatures with the package *Cosimu*. We present the properties of the pairs of signatures generated with this approach and the influence of main parameters. We illustrate how we can tune the parametrization to obtain simulated data that harbor the same amplitude and variability of real datasets. Finally, we provide benchmarks for the main tools that are widely used in signature-based drug repositioning. The *Cosimu* package is available under the GPL license at https://github.com/cgonzalez-gomez/cosimu.git.

## Materials and methods

### Aims and definitions

In our research, we integrated three distinct types of gene expression data:

(i) Per-sample expression levels: This type consists of expression levels derived from RNA-Seq read counts, which are typically normalized as counts per million. Within our manuscript, we refer to these as “base expression level,” “expression level,” or “read count.”

(ii) Between-condition differential expression signature: This data type captures the ratios of expression levels between different conditions (with multiple samples per condition), typically presented as log_2_ fold-change (LFC) values accompanied by associated *P*-values. In our manuscript, this is designated as “differential expression signatures” or simply “signatures.” It enables the identification and characterization of genes that are differentially expressed between experimental conditions.

(iii) Interconnected pair of differential expression signatures: At the center of our method, this type involves a pair of differential expression signatures, each characterized by LFC values for each gene. These signatures are linked by a connectivity score that quantifies the correlation between them. In our manuscript, we refer to these interconnected profiles as the “primary signature” and “secondary signature.”

Our focus is to simulate the interconnected pairs of differential expression profiles (iii). Our objective is to use these simulations to evaluate the effectiveness of connectivity scores. This requires generating not only the interconnected signatures but also the essential underlying data: primary signatures (ii) and base expression levels (i). When simulating these data types, we aimed to replicate the variability of realistic datasets to limit the biases of our simulations.

### Overview of the process of simulating an interconnected pair of signatures

To simulate the interconnected pair of signatures, we need to define the method for transitioning from the primary signature to the secondary. We defined the connectivity score as the parameter that influences the switching from a deregulation status (up-, down-, or non-deregulated) in the primary signature to another in the secondary signature. Therefore, we developed a method in which a differential signature is decomposed in three layers with the regulation status (up-, down-, or non-deregulated) that we refer to as the modality. Subsequently to fully encode the LFC, we defined two other layers: the sub-modality layer that represents the amplitude of the deregulation modeled with a probability density distribution and the probability value layer that is uniformly distributed and used to draw a value from the linked probability distribution function.

Hence, we defined the transition methods for each layer separately and the LFC value can be recomposed from these layers for both the primary and the secondary signatures. However, it is important to note that decomposing a given LFC value back into the three layers is not feasible as the process would yield multiple potential solutions.

### Mathematical decomposition of the gene signature into three layers

**Figure 1:**
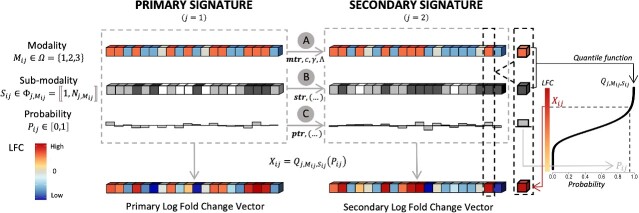
Overview of the process to simulate an interconnected pair of signatures with Cosimu. In order to simulate interconnected pairs of signatures, the LFC vector are determined by the integration of three layers: modality, sub-modality, and probability layers (represented within two frames with dotted gray borders). Following the same order of the simulation process, the first layer categorizes the genes into three modalities: up-regulated (red), down-regulated (blue), and non-deregulated (gray). Based on the modality categorization of the genes, a sub-modality is established, upon a set of N_j,Mij_ ranks that map to quantile functions associated with the probability distribution of the LFC values. The final level indicates the probability from which the exact LFC value is derived using the probability distribution function. In the right section, the process for inferring the LFC vectors from the three integration layers is depicted. Firstly, the quantile function associated with each gene is determined based on its modality category and the sub-modality rank. Subsequently, the LFC value of the gene is calculated as the image of the probability value by the quantile function. It’s important to note that this process remains consistent whether simulating the primary or the secondary LFC vector. The arrows in the central section emphasize that to simulate interconnected vectors, transitions between each layer rely on specific parameter sets. During the simulation process, we initially generate the primary LFC vector along with its corresponding layers. Following that, we simulate the secondary LFC vector based on the layers of the primary one and the parametrization of each transition. All transition details can be found in the Materials and Methods section and in Fig. S1. The final interconnected LFC vectors are depicted at the bottom, displayed as a series of cubes. Each cube symbolizes a gene, with the fill color indicating its LFC value ranging from red (up-regulated) to blue (down-regulated).

A gene (differential expression) signature can be modeled by a vector with the LFC expression values for each gene. We formulated a three-layer decomposition of such a signature into three simpler vectors. The rational argument behind this model was that each layer captured a different component of the differential expression value (Fig. 1).

Let $\Omega =\left\{1,2,3\right\}$ represent the three proposed modalities for a gene in a pairwise differential expression study: up-regulated $\left(m=1\right)$, non-deregulated $\left(m=2\right)$, or down-regulated $\left(m=3\right)$.

Given $G$ as the total number of genes where $G\in{\mathbb{N}}^{\ast }$, consider a gene $i$ (where $i\in \!\! 1,G\!\!$) and a pairwise differential expression signature $j$ (where $j\in \left\{1,2\right\}$).

$\forall m\in \Omega$ ; let ${N}_{j,m}$ indicate the number of distinct sub-modalities for modality $m$ and the signature $j$. Each sub-modality more precisely defines the amplitude of deregulation, as elaborated upon later. The collection of sub-modalities can be represented as ${\Phi}_{j,m}=\!\! 1,{N}_{j,m}\!\!$.

The LFC value, for a gene $i$ and a signature $j$, denoted as ${X}_{ij}$, is modeled through a quantile function ${Q}_{S_{ij}\mid{M}_{ij}}^j$originating from a three-layer integration showcased in Fig. 1 (and Supplementary Fig. S1) and is defined as follows:


(1) \begin{equation*} {{X}}_{{ij}}={{Q}}_{{{S}}_{{ij}}\mid{{M}}_{{ij}}}^{{j}}\left({proba}={{P}}_{{ij}}\right) \end{equation*}

with the introduced variable defined as:

-
${M}_{ij}\in \Omega$ denotes the first layer and represents the gene modality or status in a pairwise differential study.
-
${S}_{ij}\in{\Phi}_{j,{M}_{ij}}$ denotes the second layer and represents the sub-modality rank, unique to each modality, mapping to a quantile function modeling the LFC distribution and therefore its amplitude.
-
${P}_{ij}\in \left[0,1\right]$ denotes the third layer and represents the probability that determines the LFC value based on the quantile function.

This definition is highly flexible due to the potential diverse nature for the simulated signatures. While the structure remains consistent, we distinguished between two types of signatures in our approach:

$\left(j=1\right)$ The primary signature, which is independently simulated as detailed further.

$\left(j=2\right)$ The secondary signature, which is generated in connection with a primary signature using a three-layer transition model, which will be described subsequently.

#### Simulation of the primary signature $\left(j=1\right)$

The simulation of a primary signature is a three-step sampling of ${M}_{i1}$, ${S}_{i1}$ and ${P}_{i1}$ given their respective probability law given in (2, 3, 4), respectively. It is important to note that sampling of ${S}_{i1}$ is contingent upon the sampled value of ${M}_{i1}$.


(2) \begin{equation*} \forall{m}\in{\varOmega}, \mathbb{P}\left({{M}}_{{i}{1}}={m}\right)={{\omega}}_{{m},{1}} \mid \sum_{{m}}{{\omega}}_{{m},{1}}={1} \end{equation*}

(3) \begin{equation*} \forall \left({s},{m}\right)\in{{\Phi}}_{{1},{m}}\times{\Omega}, \mathbb{P}\left({{S}}_{{i}{1}}={s}\right)={{\varphi}}_{{1},{m},{s}} \mid \sum_{{s}\in{{\Phi}}_{{1},{m}}}{{\varphi}}_{{1},{m},{s}}={1} \end{equation*}

(4) \begin{equation*} \forall{i}\in \!\!{1},{G}\!\!, {{P}}_{{i}{1}}\sim{\mathcal{U}}\left({0},{1}\right) \end{equation*}

The probabilistic distributions for the modality $\left({{\omega}}_{{1},{1}}\kern0.5em {{\omega}}_{{2},{1}}\kern0.5em {{\omega}}_{{3},{1}}\right)$ and for the sub-modality, $\big\{ \big({{\varphi}}_{{1},{1},{1}}\ \dots {{\varphi}}_{{1},{1},{{N}}_{{1},{1}}}\big)\quad \big({{\varphi}}_{{1},{2},{1}}\ \dots\ {{\varphi}}_{{1},{2},{{N}}_{{1},{2}}}\big) \big({{\varphi}}_{{1},{3},{1}}\ \dots\ {{\varphi}}_{{1},{3},{{N}}_{{1},{3}}}\big)\big\}$, along with $\big\{\big({{Q}}_{{1}\mid{1}}^{{1}}\ \dots\ {{Q}}_{{{N}}_{{1},{1}}\mid{1}}^{{1}}\big)\quad \big({{Q}}_{{1}\mid{2}}^{{1}}\ \dots\ {{Q}}_{{{N}}_{{1},{2}}\mid{2}}^{{1}}\big) \big({{Q}}_{{1}\mid{3}}^{{1}}\ \dots\ {{Q}}_{{{N}}_{{1},{3}}\mid{3}}^{{1}}\big)\big\}$, their associated quantile functions, are provided as inputs to the simulation. Those parameters can be inferred from real data to build realistic datasets.

#### Transitions from the primary $\left(j=1\right)$ to the secondary signatures $\left(j=2\right)$

To generate the secondary signature based on the primary one, methods for the transition at each layer were introduced. To maintain notational clarity, the following symbols will be used and associated to gene $i\in \!\! 1,G\!\!$:

-
$\alpha \in \Omega; \tau \in{\Phi}_{1,\alpha };\psi \in \left[0,1\right]$ : are the modality, sub-modality, and probability values in the primary signature, respectively.
-
$\beta \in \Omega; \upsilon \in{\Phi}_{2,\beta };\rho \in \left[0,1\right]$ : are the modality, sub-modality, and probability values in the secondary signature, respectively.

Given a known primary signature, conditional probabilities are used to describe the sampling process. Similarly to the primary signature generation, probabilistic distributions for ${S}_{i2}$  $\left\{\left({\varphi}_{2,1,1}\ \dots\ {\varphi}_{2,1,{N}_{1,1}}\right)\ \ \left({\varphi}_{2,2,1}\ \dots\ {\varphi}_{2,2,{N}_{1,2}}\right)\\ \left({\varphi}_{2,3,1}\ \dots\ {\varphi}_{2,3,{N}_{1,3}}\right)\right\}$, along with their associated quantile functions $\big\{\big({Q}_{1\mid 1}^2\ \dots\ {Q}_{N_{1,1}\mid 1}^2\big) \big({Q}_{1\mid 2}^2\ \dots\ {Q}_{N_{1,2}\mid 2}^2\big)\quad \big({Q}_{1\mid 3}^2\ \dots\ {Q}_{N_{1,3}\mid 3}^2\big)\big\}$, are provided as inputs to the simulation.

##### Modality transition

To transition to the secondary modality layer from the primary one, a transition matrix, denoted as $\Lambda$, is defined. It signifies the conditional probabilities of observing a specific secondary modality $\beta \in \Omega$ given the primary modality $\alpha \in \Omega$:


(5) \begin{equation*} {\Lambda} ={\left({{\lambda}}_{{\alpha} {\beta}}\right)}_{\begin{array}{c}{\alpha} \in{\Omega} \\{}{\beta} \in{\Omega} \end{array}}\in{\left[{0},{1}\right]}^{{9}} \end{equation*}

where ${\lambda}_{\alpha \beta}=\mathbb{P}\big({M}_{i2}=\beta\ \big|\ {M}_{i1}=\alpha \big)$

This $\Lambda$ matrix can be given as input to the simulation. Alternatively, a theoretical model can be utilized with fewer than nine parameters. In this instance, a basic symmetric connectivity model featuring a connectivity score $(c)$ and a noise factor induced by the non-deregulated genes $\left(\gamma \right)$ such as:


$$ \forall c,\gamma \in \left[-1,1\right]\times \left[0,0.5\right], $$

$$ {\lambda}_{11}={\lambda}_{33}=\frac{1}{2}\left(1+c\right)\left(1-\frac{\gamma }{2}\right) $$

$$ {\lambda}_{13}={\lambda}_{31}=\frac{1}{2}\left(1-c\right)\left(1-\frac{\gamma }{2}\right) $$

$$ {\lambda}_{12}={\lambda}_{21}={\lambda}_{32}={\lambda}_{23}=\frac{\gamma }{2} $$

$$ {\lambda}_{22}=1-\gamma $$

##### Sub-modality transition

This transition is contingent on the modality transition. Indeed, when a gene is not deregulated in at least one signature (either when $\alpha =2$ or $\beta =2$), then, an independent transition is utilized, echoing the primary signature generation. Otherwise, more strategies are available for the transition for a total of four listed below. As stated above, $\tau \in{\Phi}_{1,\alpha }$ is the sub-modality in the primary signature, and $\upsilon \in{\Phi}_{2,\beta }$ is the sub-modality in the secondary signature,

- Independent transition

The independent transition generates a secondary sub-modality layer independently of the primary one.


(6) \begin{equation*} \mathbb{P}\left({{S}}_{{i}{2}}={\upsilon} \right)={{\varphi}}_{{2},{\beta}, {\nu}} \end{equation*}

- Deterministic transition

The deterministic transition generates a secondary sub-modality layer identical to the primary one. The transition is only feasible if both signatures possess an equal number of sub-modalities (i.e. ${N}_{1,\alpha }={N}_{2,\beta }$).


(7) \begin{equation*} {\tau} ={\upsilon} \end{equation*}

- Stochastic transition

The stochastic transition introduces inherent variability through a binomial link. Like the deterministic transition, this approach is only applicable when ${N}_{1,\alpha }={N}_{2,\beta }$.


(8) \begin{equation*} \left({{S}}_{{i}{2}}|{{S}}_{{i}{1}}={\tau} \right)\sim{\mathcal{B}}\left({n},{p}\right) \end{equation*}

with $n={N}_{1,\alpha }={N}_{2,\beta }$ the size of the sample and $p=\frac{\tau }{n}$ the success probability.

- Copula transition

The copula transition incorporates stochasticity while ensuring the marginal probabilities given as input and without requiring that ${N}_{1,\alpha }={N}_{2,\beta }$. Briefly, two dimensional copulas are used to construct a bivariate distribution that respects the assigned margins ${\left({\varphi}_{1,\alpha, \tau}\right)}_{\tau \in{\Phi}_{1,\alpha }}$and ${\left({\varphi}_{2,\beta, \nu}\right)}_{\nu \in{\Phi}_{2,\beta }}$, as well as a desired Pearson’s correlation score between the marginal distributions, denoted as $\theta$. The copula parameter is optimized with desired Pearson’s correlation as objective as described by A. Barbiero [13]. It’s worth mentioning that the implementation is valid for discrete values with underlying continuous distribution, ranked and regular intervals. Moreover, its validity depends on the input quantiles for the simulation. Three copulas function are available to the simulation: Frank, Placket, and Gauss.

The joint probabilities obtained are used to compute the conditional probabilities as follows:


(9) \begin{equation*} \mathbb{P}\big({{S}}_{{i}{2}}={\upsilon} \big|\ {{S}}_{{i}{1}}={\tau} \big)=\frac{\mathbb{P}\left({{S}}_{{i}{2}}={\upsilon} \cap{{S}}_{{i}{1}}={\tau} \right)}{{{\varphi}}_{{1},{\alpha}, {\tau}}} \end{equation*}

##### Probability transition

Finally, three probability transitions are implemented to generate the secondary probability layer based on the primary one.

- Independent transition

The independent transition generates a secondary probability layer independently of the primary one.


(10) \begin{equation*} {{P}}_{{i}\mathbf{2}}\sim{\mathcal{U}}\left({0},{1}\right) \end{equation*}

- Deterministic transition

The deterministic transition generates a secondary probability layer identical to the primary one.


(11) \begin{equation*} {\rho} ={\psi} \end{equation*}

- Copula transition

In the Copula transition, the probabilities associated with the secondary signature are derived from the primary signature based on a Copula function. Three copula functions are available for the simulation: Frank, Placket, and Gauss. Given that both marginal probability laws are fixed to $\mathcal{U}\left(0,1\right)$, the joint distribution simplifies to the conditional distribution enabling the modeling and sampling of $\rho$ conditional on a known $\psi$ as follows:


(12) \begin{equation*} \forall{\psi} \in \left[{0},{1}\right],\left({\rho}\ |\ {\psi} \right)\sim{C}\left({\theta} \right) \end{equation*}

with $C$ the Copula function $\theta$ its parameter(s).

Additionally, to the decomposition of the gene signature into three layers, we implemented a simulation pipeline to increase the likelihood of the simulated signatures (as explained in the following sections). (i) First, we generated experimental replicates using *Cosimu*. (ii) Second, the control expression levels were modeled. (iii) Third, the read counts were generated using a negative binomial model. (iv) Ultimately, differential expression signatures were re-inferred exploiting the read count matrix from the samples replicates, using DESeq2 [14] .

### Simulation of signatures replicates with *Cosimu*

**Figure 2:**
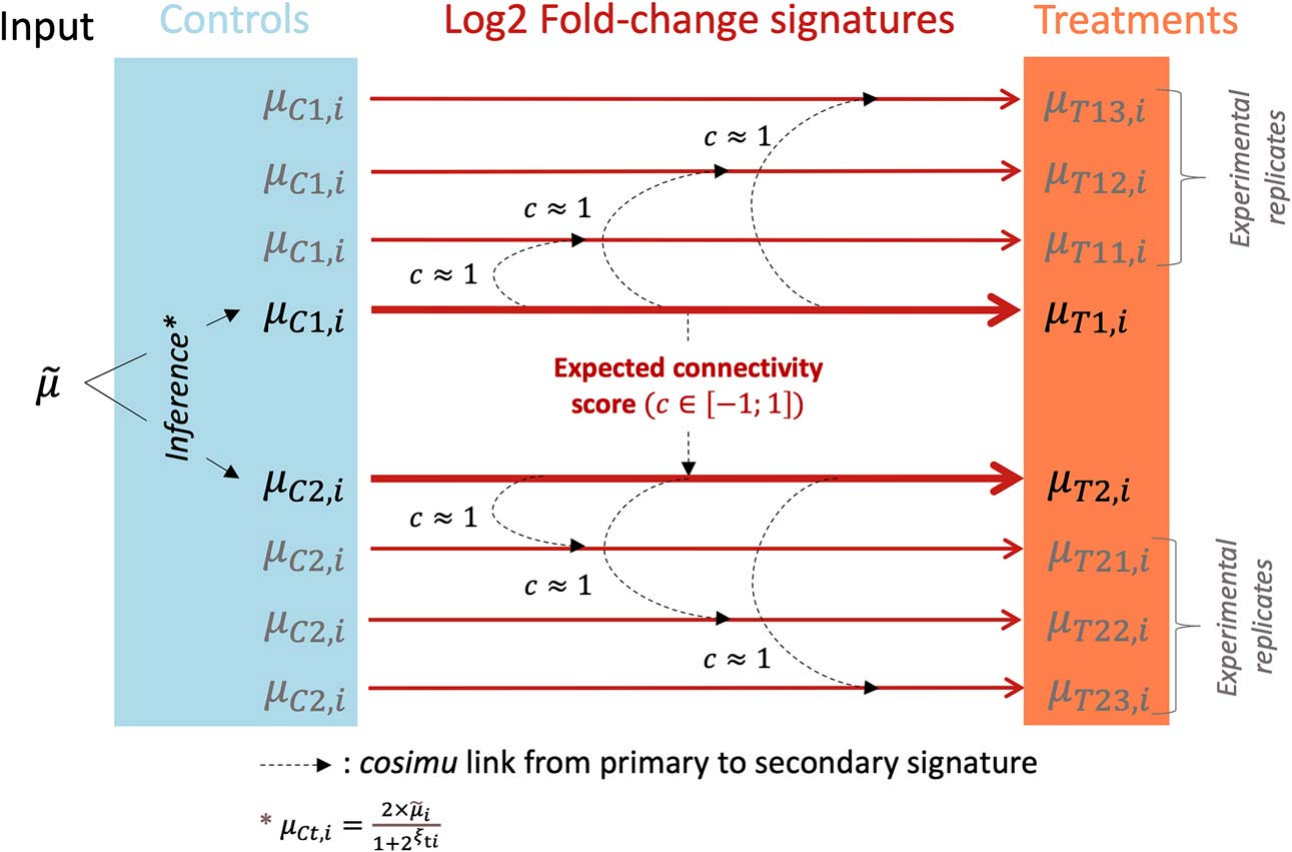
Schematic overview of the relations between treatment and control expression levels in the simulation pipeline. Additionally, beyond being inferred from the input base expression and the treatment signature, control expression levels can also have “biological” replicates (Fig. S2) generated by adding a centered Gaussian noise N(0,0.5).

*Cosimu* was also employed to generate *in silico* replicates of the simulated signatures using a connectivity parameter close to 1 and a noise factor to be fine-tuned to achieve targeted variability. This step was part of the process of simulating read counts from connected signatures with a variability close to real data (Fig. 2). During our simulations, we chose a connectivity of 0.9 and a noise of 0.02 in order to introduce some variability in the technical replicates; this was determined based on the analysis done on the impact of the parameters (Fig. 2).

### Modeling expression levels

Upon studying the properties of real datasets, we identified a significant disparity in the distribution of expression in both conditions depending on gene deregulation status: down-regulated genes were more likely to be expressed or highly expressed in the reference condition and inversely for up-regulated genes.

To reproduce this observation, we consider an input expression level ${\overset{\sim }{\mu}}_i$ (with $i\in \!\! 1,G\!\! \Big)$, which represents the mean value between both conditions: treated and control (Fig. 2). This input can be extracted from real data or generated randomly. In our simulations, we used the average normalized (TPM) gene expression from the control samples of the National Center for Biotechnology Information (NCBI) Gene Expression Omnibus (GEO) data base [15], accession number GSE185985 [16] (Supplementary Note S1). Then, the control expression level, ${\left[{\mu}_{Ct,i}\right]}_{t\in \left\{1;2\right\};i\in \!\! 1,G\!\! }$, can be computed as follows:


(13) \begin{equation*} \forall{i}\in \!\!{1},{G}\!\!, {{\mu}}_{{Ct},{i}}=\frac{{2}\times{\overset{\sim }{{\mu}}}_{{i}}}{{1}+{{2}}^{{{\xi}}_{{t}{i}}}} \end{equation*}

with ${\xi}_{ti}$ the (LFC) mean value of the sub-modality distribution associated to the ${i}^{th}$ gene (in the signature of treatment *t*  $\in \left\{1;2\right\}$).

### Read count modeling

Subsequently, we modeled the read counts by a negative binomial distribution for each treatment (1 and 2) and the associated controls:


(14) \begin{equation*} \forall \left({i},{t}\right)\in \!\!{1},{G}\!\! \times \left\{{1};{2}\right\}\ {{K}}_{{it}}\sim{NB}\left({{\Delta}}_{{it}},{{\varsigma}}_{{it}}\right) \end{equation*}

with:

- the mean ${\Delta }_{it}=\frac{\mu_{Ct,i}\times{X}_{it}\times{L}_i}{S_j}\times N$ and ${S}_t=\sum_i{\mu}_{Ct,i}\times{X}_{it}\times{L}_i$. The variables ${\mu}_{Ct,i},{X}_{it}$ and ${L}_i$ are, respectively, the control expression level, the simulated fold-change, and the length of the ${i}^{th}$ gene (set to 1 if we simulate a 3’RNA-Seq), and $N$ correspond to the total number of reads expected in each sample. Control read counts are also generated for each treatment setting ${X}_{it}={\left[1\right]}_G$.
- the dispersion factor ${\varsigma}_{ij}=\frac{\Delta _{ij}}{3}$.

### Differential expression analysis

Finally, read counts matrix were analyzed to re-infer the differential expression signatures of the treatments. The analysis were conducted utilizing the DESeq2 package [14], a well-established tool for RNA-seq data analysis. DESeq2 [14] employs negative binomial generalized linear models to assess differential expression of genes. Our approach adhered to standard practices and default parameters. We utilized count matrices as input data, comparing the two conditions—“treated” and “untreated” (with the latter serving as the reference level)—within a straightforward experimental design.

### Correlation analysis with real experimental replicates

Real count matrices were obtained from three different bulk RNA-Seq public repositories from the NCBI GEO database [15] (accession numbers GSE185453 [17], GSE181472 [18], GSE182024 [19]). Technical replicates were divided into two different groups and then analyzed using DESeq2 [14] with default parametrization. This way, we got several pairs of LFC estimations from different control/treatment conditions. Finally, the Pearson’s correlation score was estimated for each pair of LFC vectors associated to the same experiment with an expected connectivity score of 1.

### Decomposition of real data for tuning simulation parameters

To estimate the distribution of sub-modalities, we employed actual data derived from HeLa cells treated with TLN468 (accession GSE185985 [16], available at NCBI GEO database [15]). These raw data were analyzed using DESeq2 [14], and the resulting estimated LFC distribution served as the foundation for generating our simulation distributions. Afterward, we identified the modes within the actual LFC distribution and their corresponding densities, utilizing the *LaplacesDemon* package [20]. This process provided us with distribution information essential for simulating realistic data (detailed in Supplementary Note S2).

### Methods for estimating the connectivity scores

We assessed the performance of seven scores [8] developed to estimate the connectivity between differential expressed signatures. These scores included the Kolmogorov-statistic-based metrics CMAP1 [1] and the weighted connectivity score of CMAP2 [2], the connectivity strength score (CSS) [21], as well as four extreme similarity metrics (xsum, xcos, xspearman, xpearson) [5].

### Evaluation of compound retrieval performance for benchmarking of connectivity scores

Methods estimating the connectivity score between pairs of signatures have been compared through their performance to predict a drug mode of action [4]. Cheng *et al*. introduced the use of the area under the curve (AUC) of the Receiver Operating Characteristic (ROC) for connectivity-based method evaluation. This score has been extensively re-used in other studies aiming at evaluating connectivity-based screening methods [6, 9, 12, 22]. In those studies, the AUC was computed at a given false discovery rate assuming it is acceptable to sacrifice some true positive to keep false positive low [4]. Nevertheless, the whole pre-clinical development cost is less than the clinical-stage costs [23], and in addition, a pre-clinical validation of an *in silico* candidate is only a fraction from the preclinical costs and time, especially with recent advances in biological model development [24]. Hence, it could be more advantageous to identify many hits at a pre-clinical stage. On top of that, it would increase the number of eligible candidates for the selection of a lead that would have the best chance of success in the latter clinical development [25] considering medical and economic contexts. In other words, even if the ROC is adapted to a binary problem such as the retrieval of at least one compound, the precision–recall curve is more adapted for problems requiring the retrieval of a maximum of true positive at a minimum cost [26].

For benchmarking the seven previously presented connectivity scores, the objective was to assess the capacity of each method to identify true positive interactions from a pool of signatures (based on the estimated connectivity scores). This task closely resembled the field of information retrieval (IR) systems. Indeed, in IR, the objective is to retrieve relevant documents or information from a large pool of non-relevant items, given a query. Therefore, to evaluate the scores, we employed a well-established metric in the field of IR: the average precision, which is based on precision–recall curves [27, 28].

Let the precision function $p$ be a function of the recall; the average precision for a method $\lambda$ is computed as follows: 


(15) \begin{equation*} {{AP}}_{{\lambda}}={\int}_{{0}}^{{1}}{p}\left({x}\right){dx} \end{equation*}

which corresponds to the area under the precision–recall curve, but in practice, it is calculated with a finite sum over every position in the ranked sequence of retrieved items: 


(16) \begin{equation*} \overset{\sim }{{{AP}}_{{\lambda}}}=\sum_{{k}={1}}^{{n}}{{P}}_{{k}}\Delta{{r}}_{{k}}=\sum_{{k}={1}}^{{n}}{{P}}_{{k}}\left({{r}}_{{k}}-{{r}}_{{k}-{1}}\right) \end{equation*}

where ${P}_k$ is the precision at cut-off $k$ in the list and $\Delta{r}_k={r}_k-{r}_{k-1}$ is the difference in the recall from items $k-1$ to $k$.

## Results

In our study, we developed a method for simulating datasets that include pairs of interconnected signatures. This process involves modeling each signature as LFC vector, which is generated by the integration of three layers: modality, sub-modality, and probability layers, as detailed in Fig. 1 and Supplementary Fig. S1. The complete set of parameters used in our analyses is provided in the supplementary materials (Supplementary Tables S1 and S2).

### Properties of the simulated data

**Figure 3:**
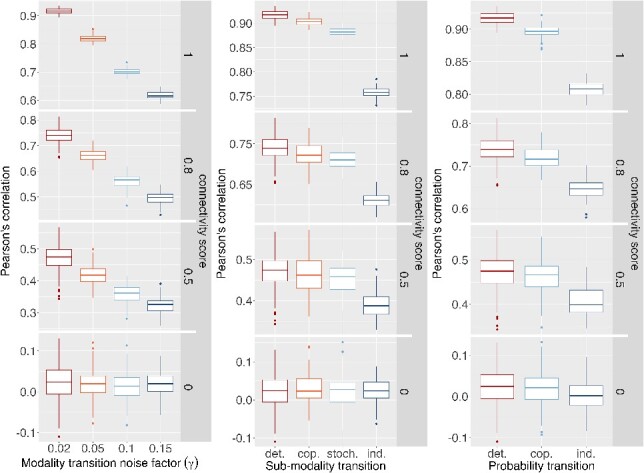
Influence of the transition parametrizations on Pearson’s correlation–based connectivity score estimation. Simulated data generated with varied modality, sub-modality, and probability transitions parameters. Reference state: γ = 0.02; sub-modality transition: “deterministic”; probability transition: “deterministic.” Additional parametrization details in Supplementary Table S3.

Initially, we examined the fundamental characteristics of the simulated signatures by calculating the Pearson correlation coefficient for each pair (primary and secondary signatures derived from the primary). We observed similar outcomes when employing the Spearman correlation. Notably, as the connectivity between signatures was nullified and the noise factor increased, the variability in correlation scores decreased (Fig. 3A). This trend could be anticipated since higher noise reduces the likelihood of value similarities when across distinct modalities. Furthermore, an increase in the noise led to a shift of the correlation scores towards zero. Importantly, when the noise factor was minimal, the average correlation score approached the predefined connectivity value.

Figure 3B highlights the effects of different transition types on the sub-modality layer, showing that non-deterministic transitions (such as copula and stochastic transitions) caused only minor shifts in the mean correlation score compared to the significant shift resulting from independent transitions. Similarly, the impact of transition types applied to the probability layer is shown in Fig. 3C, with the shift induced by stochastic transitions lying between those caused by deterministic and independent transitions. This analysis underscored the capability of our simulation method to generate diverse gene expression signatures with controllable levels of correlation and noise. ‘Stochastic’ and ‘copulas’ transitions served as an intermediary level through which we could introduce noise while maintaining the relation between the primary and the secondary layers. It is important to notice that even if these transition factors modulate the estimated score, they do not create a chimeric correlation when the signatures are not connected (null connectivity score).

### Data augmentation: from real datasets to realistic simulated data

During the development of this versatile simulation tool, a critical task was to identify the optimal parameter set that best captured the variability observed in real biological data (see Materials and Methods and Supplementary Note S2).

**Figure 4:**
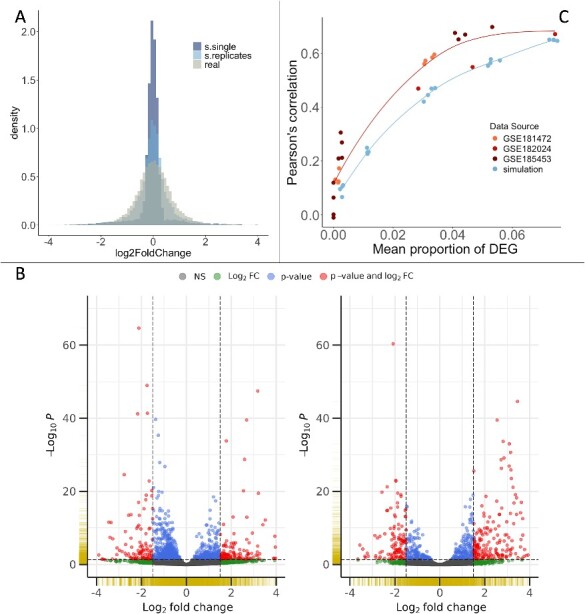
Comprehensive comparison between the simulated and real data characteristics. (A) Histogram comparison between simulated and real data. The plot depicts “s.single” and “s.replicates,” representing simulated data before and after incorporating the experimental variability. (B) Volcano plots. The left plot corresponds to real data, while the right one represents simulated data. Both datasets were analyzed using a non-paired design. Adjusted *P*-values were obtained using the Benjamini and Hochberg method and filtered at −log10P < 70. (C) Correlation comparison. Pearson’s correlation score was calculated between 23 pairs of real LFC signatures, each one inferred from the DESeq2 differential expression analysis from different experimental replicates (three controls versus three treatment). The proper was done with 20 pairs of interconnected simulated signatures. The last were simulated with variable proportions of deregulated genes, a connectivity of 1, “deterministic” transitions, and a noise equal to 0.02 for biological replicates and interconnected signatures. For the read counts generation size factor of 0.7 was selected, which makes the negative binomial distribution to converge to a Poisson distribution that captures technical variability [33]. The regression curves were determined using the function geom_smooth [34]. Kruskal–Wallis test was run to evaluate if the medians of the estimated correlation scores were identical across the three levels of DEG proportions (low < 0.02, medium, and high 0.04). The results for real data (H (2) = 17.464, *P* = 1.6e-4 <.05) and simulated data (H (2) =16.457, *P* = 2.7e-4< .05) allowed us to reject the null hypothesis.

As described in the methods, the aim was to find a parametrization that reproduced the amplitude and variability of real datasets. One key element was the calculation of the control expression levels at the basis of read count simulation (as detailed in Materials and Methods section). Figure 4 illustrates the importance of considering the input expression levels as the mean value of both condition (treatment and control) as well as the importance of introducing experimental variability through the generation of replicates, read counts and the differential expression analysis (Fig. 2). Indeed, before the introduction of the variability, there is a peak around 0 in the simulated distribution. By reproducing the pipeline used to generate the real RNA-Seq data, this peak is flattened and the distribution of the simulated LFC is closer to the one from real data (Fig. 4A). On the other hand, if we compare the two volcano plots generated with simulated data (Fig. S5), we can see that without the inference of the control base expression (Equation (13)), there is an overestimation of the *P*-values from the up-regulated genes. This trend is an artefact caused by the absence of link between the simulated LFC signatures and the input expression level. When the base expression is also modeled, the final signature distribution is more balanced and behaves similarly to real data (Fig. 4B).

We used real datasets to setup the simulation parameters as described in the methods (Supplementary Note S2 and Fig. S3). Our objective with these simulations was to mirror the actual data’s variability (reflected in *P*-value distributions) and the amplitude of deregulation (distribution of LFC values). Through a volcano plot analysis (Fig. 4B), we show that our refined set of parameters effectively captures the variability and LFC distribution characteristics of genuine RNA-Seq differential expression signatures. To quantify the degree of similarity between real and simulated data we calculate the Kullback–Leibler (KL) divergence. A KL divergence of 0 indicates that both distributions are identical, and we used as reference the divergence between the signatures inferred from two experimental replicates and obtain an average score of 0.12. The KL divergence between the distribution of the simulated data and the real one (from which the parametrization was inferred) was equal to 0.19, while it reached 0.65 between two independent real signatures (Supplementary Table S6).

Further validation of our simulation approach involved comparing Pearson’s correlation scores between pairs of connected signatures from both simulated samples and real datasets (Fig. 4C). This comparison aimed to assess whether the correlation scores derived from simulated data fell within a realistic range when compared to those obtained from actual experiments. Given that these datasets are expected to have a connectivity of 1, because they are biological replicates, any observed lower correlation scores would be attributed to noise. Our goal was to determine if the simulation could accurately replicate the noise patterns observed in real data, using an optimized set of parameters. We then generated different simulated datasets with an expected connectivity score of 1 and the parametrization inferred from real-data distribution (‘Primary Signature’ section of Supplementary Table S5) while varying the global proportion of deregulated genes. The dependency observed between the proportion of differential expressed genes (DEG) and the estimated correlation between biological replicates is conserved on the simulated interconnected signatures, which was confirm by a Kruskal–Wallis test. This suggests that our simulations, at least when aiming for extreme connectivity scores, replicates the dependencies and noise pattern of real datasets.

### Benchmarking compound retrieval performance

One of our goals by simulating interconnected differential expressed signatures was to be to offer a homogenic way to evaluate the different connectivity scores that currently exist in the literature. For this aim, we exploited the previous parametrization to simulate the interconnected signatures. Precisely, we used the sub-modalities distributions and proportions inferred from real data, an adapted non-deregulated noise $\left(\gamma =0.02\right)$, and 500 connectivity scores $c\in \left[0;1\right]$ (Supplementary Tables S4 and S5) to simulate our evaluation data. Subsequently, we conducted a paired differential expression analysis, based on the simulated triplicates, to estimate the expression signatures while accounting for the introduced inference bias.

All the pairs of interconnected signatures were then given as input to the seven evaluated methods, for them to estimate the connectivity score associated to each couple. The pairs were then ordered by decreasing connectivity score. This ordered list was then compared to the expected results based on the connectivity scores that were used to simulate the interconnected signatures, in order to assess the performances of the different methods. For this purpose, the average precision score, an evaluation metric commonly employed in IR systems, was employed.

**Figure 5:**
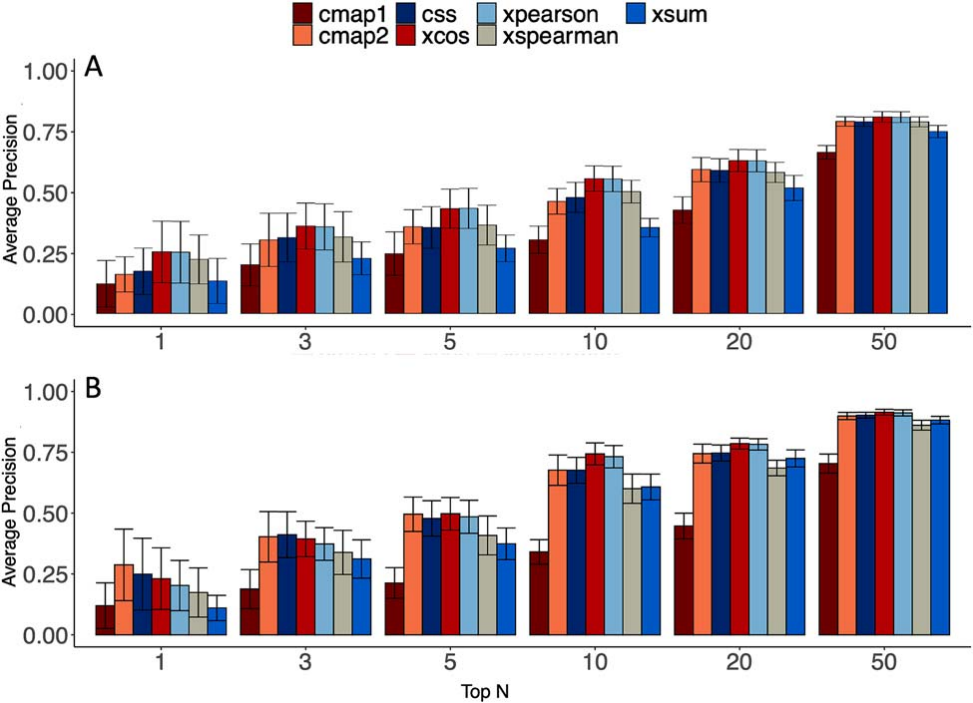
Average precision score as a function of the number of positively labeled signatures in the evaluation dataset (top *N*). Mean of the average precision scores were calculated over 20 replicates, and the error bars represent the 95% confidence interval. (A) Dataset fully inspired from real-data distribution, see Table S4. Dataset generated with asymmetrical deregulated genes proportions (p_up = 0.07 and p_down = 0.13) and noise factor (nr_noise) of 0.02. (B) Dataset inspired from an averaged realdata distribution (Table S5) except for the deregulated genes proportions (p_up = 0.05 and p_down = 0.15) and noise factor (nr_noise) of 0.02.

The results of this evaluation are shown on Fig. 5, with the comparative visualization of the estimated average precision scores. It shows that extreme cosine similarity and Pearson correlation consistently outperform other scores on this dataset, particularly when evaluating the retrieval of a limited number of signatures of interest (top *N* < 10). Furthermore, extreme Spearman correlation performs at least as well as CMAP2 and CSS, specially detecting the top 1. Contrary to what was found previously [5], the extreme sum similarity score surpasses the performance of the original CMAP1 score but not the other metrics in the evaluated samples.

Supplementary results (Fig. S4), generated with a symmetrical distribution, cover various types of parametrizations including different proportions of deregulated genes and the introduction of non-deregulated noise. This additional benchmarking revealed new insightful observations. In particular, XSpearman appears to be more sensitive to the imbalance on the deregulated proportions compared to XSum. Furthermore, among the top four scores, XCos emerges as the most susceptible to the influence of non-deregulated noise.

**Figure 6:**
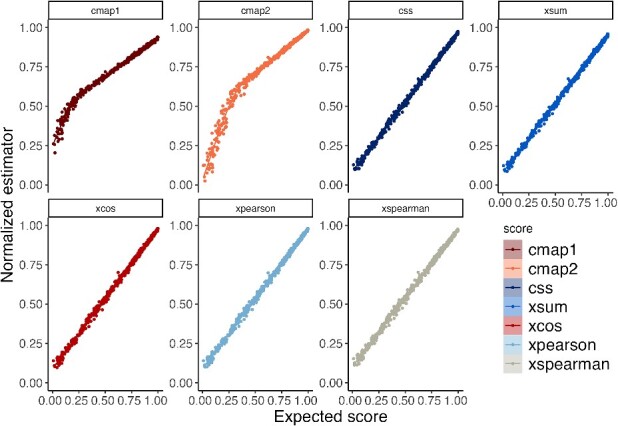
Regression of the connectivity scores estimations. Regression analysis was performed using generalized additive models (GAM), and the Bayes credible interval was calculated using the function geom_smooth [34].

In addition to the average precision score evaluation, we conducted a regression analysis (Fig. 6) between the estimated normalized scores and the theorical connectivity score used to generate the simulated pair of signatures. Notably, the two Kolmogorov-based methods are the only ones that lack a linear relation. Instead, two distinct linear trends are evident: one for low connectivity scores and another one for the higher values. Interestingly, the scores with lower average precision scores exhibit less pronounced slopes in their linear trends.

## Discussion

### Understanding the simulation process and its parametrization

Analyzing the properties of the simulation method is essential for determining how to appropriately parameterize the algorithm to generate datasets with the desired characteristics. As highlighted by Morris *et al*. [29], the choice of the data-generating mechanism is crucial during a simulation study; it corresponds to the second stage of the “ADEMP” approach [29]. In this context, we propose a simulation-based method that provides considerable flexibility in terms of parametrization, underscoring the necessity for users to have clear objectives before initiating the simulation process. We recognize the significance of this aspect, as emphasized in Ripley’s [30] work, where conclusions drawn from the analysis of simulated data can be prone to misinterpretation. Therefore, careful consideration and adherence to predefined aims are imperative to ensure the validity and reliability of simulated results.

The comparison of the effects arising from the diverse parametrizations on connectivity score estimations substantiated the expected outcomes based on the mathematical definitions. On one hand, the influence of the non-deregulated noise parameter was readily predictable from the definition of the transition matrix (Equation (5)). The noise level exhibits an inverse relation with the estimated correlation, directly impacting the degree of connectivity between signatures. This is likely because transitions involving non-deregulated genes are independent, which noises the relation between layers.

On the other hand, the stochastic transition offers an intermediate level of variation between the pair of signatures that is reflected on the estimation of the connectivity score. Additionally, the utilization of copulas permits the simulation of sub-modality and probability vectors that conform to the constraints of correlation and proportions [13]. This functionality controls aspects that were previously impossible to constrain, except through a deterministic transition (and only in the case where the proportions were identical between the primary and secondary signatures).

### Implications of data augmentation through empirical parametrization

In contemporary research, simulation algorithms have become a prevailing approach for closely replicating real-world data responses. Notably, user simulation plays a crucial role in evaluating information access systems [31]. Despite the inherent challenge of accurately mimicking real data, researchers increasingly rely on simulated data for both system evaluation and training. To achieve this, rigorous validation of certain conditions is essential. Moreover, simulation data provide a unique opportunity to generate theoretically possible scenarios that are rarely observed in real-world contexts.

For the simulated datasets presented in the paper, the gamma distribution family was selected to model deregulated sub-modalities, while the Gaussian distribution was chosen for the non-deregulated one. This combination of multimodal distributions was used to capture the shape of a real data distribution, although with inherent limitations, particularly near zero.

The objective behind tuning the simulation parameters was to infer them in a non-arbitrary manner, grounded in real data, rather than to replicate real data exactly. The efficacy of this strategy is highlighted by our results: we obtained volcano plots that could be juxtaposed with real counterparts, showcasing coherent *P*-values and consistent LFC value ranges, along with low KL divergence, indicating the close resemblance between real and simulated LFC distributions. Additionally, the Pearson’s correlation scores between pairs of simulated samples align closely with those obtained from real experimental replicates.

### Interpretation of the connectivity scores evaluation

The benchmarking across seven different connectivity scores highlighted the top four: the extreme similarity scores XCos and XPearson, the weighted connectivity score (CMAP2), and the CSS. Notably, these scores also persisted as the top four performers across various datasets simulated with distinct characteristics (Supplementary Fig. S4). XSpearman and XSum secured the fifth and sixth positions, but their average precision scores significantly surpassed that of CMAP1, which had the lower results across all datasets.

In scenarios where only gene outcome information (up- or down-regulated) is available for the query signature, the employment of XSum is preferred over CMAP1, as corroborated by J. Cheng and L. Yang paper [5]. However, it’s worth noting that CMAP2 wasn’t evaluated in this study. In our present benchmark, CMAP2 consistently outperforms the XSum, highlighting the advantageous nature of GSEA’s signed two-sample Kolmogorov statistic over the “simple” [5] XSum score.

In cases where gene expression data of the query signature are accessible, the most effective scores are XPearson and XCos, both of which are parametric methods. CMAP2, CSS, and XSpearman are non-parametric scores, based in gene expression ranks. While these are suitable for heterogeneous contexts, they entail a loss of information. In our simulated datasets, the signatures lack of heterogeneity, usually present when query signatures and profiles are generated from different biological models (e.g. distinct cellular lineages), which could account for the observed performance.

This leaves CMAP1 as the sole choice when only gene membership is available for the profiles signature, and the quality and/or homogeneity of profile signatures are suboptimal—which corresponds to the case of the initial version of the CMAP database [1].

Interestingly, we have shown that performances can depend on the characteristics of the primary signature such as the proportions of deregulated genes. Hence, we might use this tool to evaluate the best score depending on the characteristics for a given disease signature prior to a screening for drug repurposing using connectivity. Nevertheless, our method does not account for the gene inter-dependencies and we cannot evaluate network-based approaches for connectivity-based repurposing.

In various fields such as biomechanics, similar tools to *Cosimu* have been developed for the generation of evaluation data. For instance, the application *OpenSim* [32] provides an open-source simulation algorithm facilitating the study of movement through simulation. This tool serves as a foundational platform for evaluating and developing new applications and making discoveries across domains such as mechanics, robotics, and neuroscience. Although our tool is currently less mature, it represents an innovative approach to studying transcriptomic interactions, at a low computational cost (Supplementary Note S3). We anticipate that it could help scientists to pick the method adapted for their data and therefore improve the predictivity of their connectivity-driven drug repurposing.

**Key Points**

- *Cosimu* is a novel simulation tool for interconnected differential expression signatures that grants users the flexibility to tailor data properties, to best mimic real data they could obtain on their research.
- Its primary objective is to facilitate the benchmarking of methods that estimate connectivity scores. We detailed an illustrative application where parameters were inferred to reproduce the real data available in terms of variability and amplitude.
- Results of this study already provide valuable insights for the scientific community, helping to gain a better understanding of which scores perform better for their specific applications.
- Notably, connectivity scores based on extreme Pearson’s correlation and cosine similarity exhibit best performance in our evaluation exercises. Nevertheless, it’s important to recognize that outcomes can be highly influenced by the properties of the simulated data.

## Supplementary Material

SUPPLEMENTARY_bbae299

## Data Availability

The authors confirm that the data supporting the findings of this study are available within the article and its supplementary materials. Package and code are available on https://github.com/cgonzalez-gomez/cosimu.git and supplementary scripts on *Cosimu* application are available on https://github.com/cgonzalez-gomez/cosimu_application.git.

## Author Biographies

**Catalina Gonzalez Gomez** received an engineer degree in Bioinformatics and Modelling from INSA Lyon in 2021. She is currently a second-year PhD candidate at the University Claude Bernard Lyon 1. Her research interests include biostatistics, machine learning, and molecule effect prediction.

**Dr Manuel Rosa-Calatrava** is a professor at Institut National de la Santé et de la Recherche Scientifique, Principal Investigator, and Team leader at Centre International de Recherche en infectiologie, Lyon, France, and an associated professor at Université Laval, Québec, Canada. He is a well-known expert in viral infectious respiratory diseases, with a strong focus on innovative antivirals and vaccine development.

**Dr Julien Fouret** received his PhD degree in Comparative Phylogenomics from the École Normale Supérieure de Lyon in 2018. He was head of data science at Signia Therapeutics from 2019 to 2023 focusing on *in silico* drug screening. From 2024, he is head of R&D at Nexomis, a bioinformatic research platform at Université Lyon 1.
