# Supplementary material for: Optimizing in silico drug discovery: simulation of connected differential expression signatures and applications to benchmarking
Source: Brief Bioinform. 2024 Jun 27;25(4):bbae299. doi: 10.1093/bib/bbae299 (PMC11210109; doi:10.1093/bib/bbae299)
Supplement: SUPPLEMENTARY_bbae299 [file supplementary_bbae299.docx]

# SUPPLEMENTARY

Figure S1 : Developed scheme of the tree layers decomposition


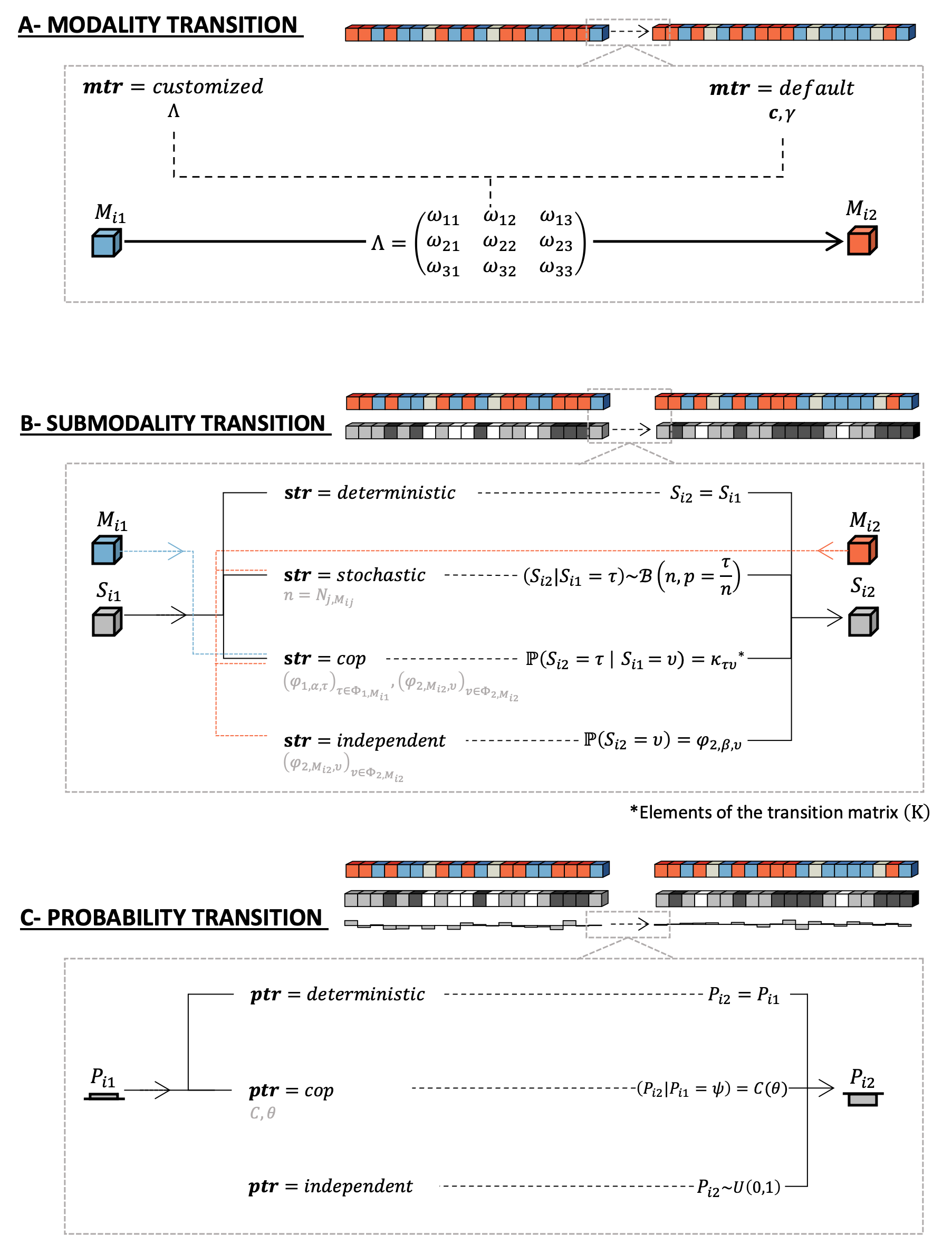


The transitions illustrated in this figure are detailed in the Materials and Methods section. To simulate the interconnected log fold-change (LFC) vectors, it is necessary to establish relations that connect the layers of the secondary signature and those of the primary one. To achieve this, we implemented various transition methods for each layer, as depicted in the figure. Each transition requires specific input elements, which may include parameters as well as values associated to the same gene but from another layer and/or the primary signature.

Table S1: Parameters description of the Primary signature

| **NOTATION** | **PACKAGE** | **RANGE** | **DESCRIPTION** |
| --- | --- | --- | --- |
| $G$ | nb_ent | $\mathbb{R}^{*+}$ | Number of genes to be simulated. |
| Modality layer | | | |
| $\omega_{1}$ | p_up | $\left[ 0;1 \right]$ | Proportion of up regulated genes. |
| $\omega_{3}$ | p_down | $\left[ 0;1 \right]$ | Proportion of down regulated genes. |
| Sub-modality layer | | | |
| $\left[ \varphi_{1,1,s} \right]_{s\in\Phi_{1,1}}$ | prop_sm_up | $\left[ 0;1 \right]^{N_{1,1}}$ | Vector of $\left( N_{1,1} \right)$ proportions for the sub-modalities of the up modality. |
| $\left[ \varphi_{1,3,s} \right]_{s\in\Phi_{1,3}}$ | prop_sm_up | $\left[ 0;1 \right]^{N_{1,3}}$ | Vector of $\left( N_{1,3} \right)$ proportions for the sub-modalities of the down modality. |
| Probability layer | | | |
| $\left[ Q_{1,1,s} \right]_{s\in\Phi_{1,1}}$ | qf_vect_up | $\left[ f:\left[ 0;1 \right]\mathbb{\to R} \right]^{N_{1,1}}$ | Vector of quantile functions associated to the up regulated genes. |
| $Q_{1,2,1}$ | qf_vect_nr | $f:\left[ 0;1 \right]\mathbb{\to R}$ | Vector of quantile functions associated to the non-deregulated genes. |
| $\left[ Q_{1,3,s} \right]_{s\in\Phi_{1,3}}$ | qf_vect_down | $\left[ f:\left[ 0;1 \right]\mathbb{\to R} \right]^{N_{1,3}}$ | Vector of quantile functions associated to the down regulated genes. |

Table S2: Parameters description of the Secondary signature

| **NOTATION** | **PACKAGE** | **RANGE** | | | | | | **DESCRIPTION** |
| --- | --- | --- | --- | --- | --- | --- | --- | --- |
| Modality layer | | | | | | | | |
| $\left[ M_{1i} \right]_{i\in\left⟦ 1;G \right⟧}$ | prim_mod  _vect | $\left\{ 1,2,3 \right\}$ | | | | | | Primary modality vector. |
| - | mod  _transition | customized | | | default | | | Modality transition. |
| $c$ | connectivity  _score | - | | | $\left[ -1;1 \right]$ | | | Expected connectivity score between the pair of signatures. |
| $\gamma$ | nr_noise | - | | | $\left[ 0;0.5 \right]$ | | | Noise induced by the non-deregulated genes. |
| $\Lambda$ | transition  _matrix | $\left[ 0;1 \right]^{3\times3}$ | | | - | | | Modality transition matrix. |
| Sub-modality layer | | | | | | | | |
| $\left[ S_{1i} \right]_{i\in\left⟦ 1;G \right⟧}$ | prim  _submod  _vect | $\mathbb{N}^{*}$ | | | | | | Primary sub-modality vector. |
| - | submod  _transition | independent | stochastic | | cop | | dependent | Sub-modality transition. |
| - | copula_sm | - | - | | {Gauss; Plackett; Frank} | | - | Copula family. |
| - | rho | - | - | | $\left[ -1;1 \right]$ | |  | Expected correlation between the deregulated sub-modalities vectors. |
| - | eps_sm | - | - | | $\mathbb{R}^{*+}$ | | - | Tolerance in the copula parameter optimization. |
| $\left[ \varphi_{2,1,s} \right]_{s\in\Phi_{2,1}}$ | prop_sm  _up | $\left[ 0;1 \right]^{N_{2,1}}$ | - | | $\left[ 0;1 \right]^{N_{2,1}}$ | | - | Vector of $\left( N_{2,1} \right)$ proportions for the sub-modalities of the up modality. |
| $\left[ \varphi_{2,3,s} \right]_{s\in\Phi_{2,3}}$ | prop_sm  _up | $\left[ 0;1 \right]^{N_{2,3}}$ | - | | $\left[ 0;1 \right]^{N_{2,3}}$ | | - | Vector of $\left( N_{2,3} \right)$ proportions for the sub-modalities of the down modality. |
| Probability layer | | | | | | | | |
| - | proba  _transition | independent | | cop | | dependent | | Probability transition. |
| - | copula_prob | - | | {Gauss; Plackett; Frank} | | - | | Copula family. |
| - | theta_prob | - | | $\mathbb{R}$ | | - | | Copula parameter^1^ |
| - | nbins_prob | - | | $\mathbb{R}^{*+}$ | | - | | Number of bins for copula function |
| $\left[ Q_{2,1,s} \right]_{s\in\Phi_{2,1}}$ | qf_vect_up | $\left[ f:\left[ 0;1 \right]\mathbb{\to R} \right]^{N_{2,1}}$ | | | | | | Vector of quantile functions associated to the up regulated genes. |
| $Q_{2,2,1}$ | qf_vect_nr | $f:\left[ 0;1 \right]\mathbb{\to R}$ | | | | | | Vector of quantile functions associated to the non-deregulated genes. |
| $\left[ Q_{2,3,s} \right]_{s\in\Phi_{2,3}}$ | qf_vect_down | $\left[ f:\left[ 0;1 \right]\mathbb{\to R} \right]^{N_{2,3}}$ | | | | | | Vector of quantile functions associated to the down regulated genes. |

^1^Copula parameter $(\theta)$ determines the characteristics of the copula function and the relation that it models:

- Gauss copula : $\theta\in[-1;1]$
  - $\theta\to1$ : comonotonicity
  - $\theta\to0$ : independency
  - $\theta\to-1$ : counter monotonicity
- Plackett copula : $\theta\in\mathbb{R}\backslash\{1\}$
  - $\theta\to$ +∞: comonotonicity
  - $\theta\to1$ : independency
  - $\theta\to0$ : counter monotonicity
- Frank copula : $\theta\in\mathbb{R}$
  - $\theta\to$ +∞ : comonotonicity
  - $\theta\to0$ : independency
  - $\theta\to$ -∞ : counter monotonicity

Table S3: Parametrization for analyzing simulation algorithm properties

| **PARAMETERS** | **VALUES** | | | | |
| --- | --- | --- | --- | --- | --- |
| nb_ent | 10000 | | | | |
| PRIMARY SIGNATURES | | | | | |
| MODALITY LAYER | | | | | |
| p_up | 0.05 | | | | |
| p_down | 0.05 | | | | |
| SUBMODALITY LAYER | | | | | |
| prop_sm_up | {0.4; 0.35; 0.25; 0.1} | | | | |
| prop_sm_down | {0.4; 0.35; 0.25; 0.1} | | | | |
| PROBABILITY LAYER | | | | | |
| qf_vect_up | $\Gamma$(*k*, 0.3) with *k* $\in$ {4; 7; 12; 15} | | | | |
| qf_vect_down | $-\Gamma$(*k*, 0.3) with *k* $\in$ {4; 7; 12; 15} | | | | |
| qf_vect_nr | $\mathcal{N}$(0, 0.15) | | | | |
| SECONDARY SIGNATURES | | | | | |
| MODALITY LAYER | | | | | |
| connectivity_score | $\{0; 0.5; 0.8; 1\}$ | | | | |
| mod_transition | default | | | | |
| nr_noise | 0.02 | | | | |
| SUBMODALITY LAYER | | | | | |
| submod_transition | independent | stochastic | cop | | dependent |
| copula_sm | - | - | Frank | | - |
| rho | - | - | 0.9 | | - |
| eps_sm | - | - | 0.001 | | - |
| prop_sm_up | {0.4; 0.35; 0.25; 0.1} | - | {0.4; 0.35; 0.25; 0.1} | | - |
| prop_sm_down | {0.4; 0.35; 0.25; 0.1} | - | {0.4; 0.35; 0.25; 0.1} | | - |
| PROBABILITY LAYER | | | | | |
| proba_transition | independent | cop | | dependent | |
| copula_prob | - | Frank | | - | |
| theta_prob | - | 10 | | - | |
| nbins_prob | - | 100 | | - | |
| qf_vect_up | $\Gamma$(*k*, 0.3) with *k* $\in$ {4; 7; 12; 15} | | | | |
| qf_vect_down | $-\Gamma$(*k*, 0.3) with *k* $\in$ {4; 7; 12; 15} | | | | |
| qf_vect_nr | $\mathcal{N}$(0, 0.15) | | | | |

Note S1: Inference of basal signatures

Read counts at gene level are normalized with a TPM intra-normalization method. Normalized read counts are then averaged by gene, generating an average expression vector. This vector serves as base for the control gene expression signature, if the bias correction is needed the vector will be modified (will be the input, see Materials and Methods).

Figure S2: Schematic overview of biological replicates for control expression levels


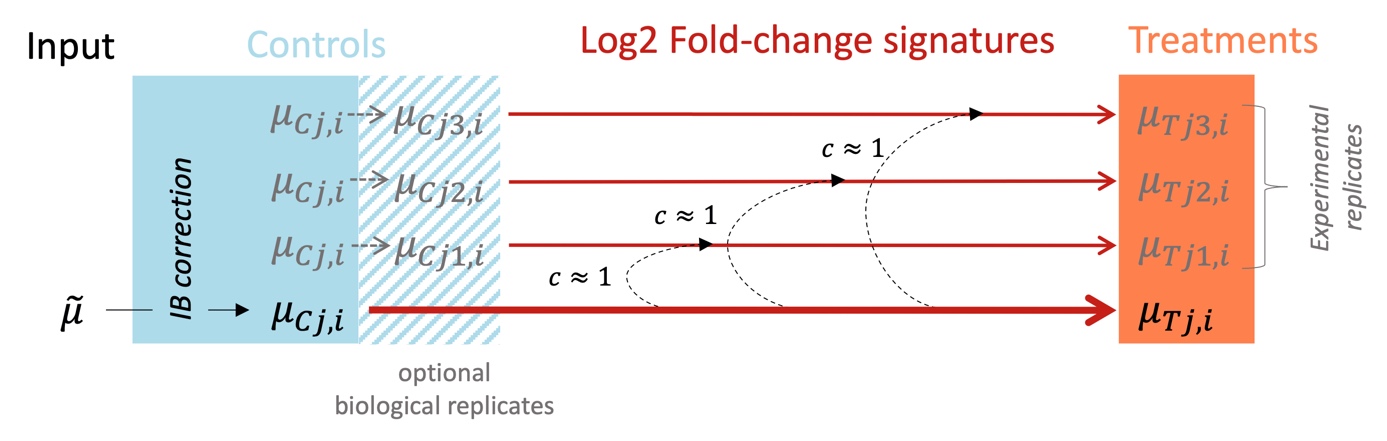


Note S2: Parametrization of the primary signature from real data

First, we started by running a differential expression analysis on the real data, using DESeq2 package^[[1]](#footnote-2)^ with the default parametrization. The log2 fold-change distribution was then evaluated for unimodality using LaplacesDemon package^[[2]](#footnote-3)^, and the modes with a minimal size of 0.01 were determined. The minimal size was fixed in order to have enough modes (not unimodal distribution) but also modes with a considerable density, we used the simultaneous visualization of the log2 fold-change distributions and the identified modes to determine this parameter. The function Modes of the LaplacesDemon package^[[3]](#footnote-4)^, return the mode value and its density among other information.

The modes were then classified in three categories: up (>0.1), non-deregulated and down (<0.1). This classification was done with symmetric thresholds around 0, fixed at 0.1 and -0.1, the number of modes in each one of the deregulated categories (up and down) determine the number of sub-modalities $(N)$. We consider that a multigamma distribution is a good fit for the log2 fold-change values on the deregulated modalities. We fixed their scale $(\theta=0.2)$ and then average the contiguous modes $(m)$ to determine the shape $(k)$ of the distributions of each sub-modality.

$$k_{i}=\left\{ \begin{aligned} \frac{{(m}_{i}+m_{0})}{2\theta}+1, \text{if }i=1 \\ \frac{{(m}_{i}+m_{i-1})}{2\theta}+1, \text{ else} \end{aligned} \right., i\in\left⟦ 1,N \right⟧$$

With $m_{0}$ the maximal (for up sub-modalities) or minimal (for down sub-modalities) mode value of the non-deregulated category.

Similarly, we determined the density for each sub-modality in order to be able to calculate their proportions for the simulation. To calculate the densities (d) we averaged the densities of the contiguous modes.

$$d_{i}=\left\{ \begin{aligned} d_{i}+d_{0}, \text{if }i=1 \\ d_{i}+d_{i-1}, \text{ else} \end{aligned} \right., i\in\left⟦ 1,N \right⟧$$

Finally, we used the sum of the submodalities densities to determine the proportions of each categorie. With this protocol we determine all the parametrization for the deregulated modalities, the distribution of the non-deregulated modality is gaussian. We fixed the mean at 0 and the standard deviation was ajusted manually, by grid search in the intervall [0.05, 0.2].

Figure S3: Log2 Fold-Change simulation distribution, parametrized based on real data distribution.


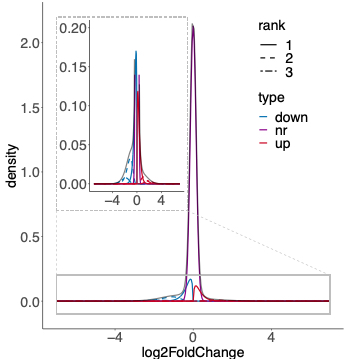
Distribution employed for data simulation, parameters were inferred following the Protocol S2.

Table S4: Parameters inspired of real data distribution for benchmarking of connectivity scores

| **PARAMETERS** | **VALUES** |
| --- | --- |
| nb_ent | 19338 |
| PRIMARY SIGNATURES | |
| MODALITY LAYER | |
| p_up | 0.07 |
| p_down | 0.13 |
| SUBMODALITY LAYER | |
| prop_sm_up | {0.777; 0.145; 0.078} |
| prop_sm_down | {0.610; 0.301; 0.089} |
| PERCENTILE LAYER | |
| qf_vect_up | $\Gamma$(*k*, 0.2) with *k* $\in$ {4.92; 10.40; 12.90} |
| qf_vect_down | $-\Gamma$(*k*, 0.2) with *k* $\in$ {1.67; 6.32; 10.06} |
| qf_vect_nr | $\mathcal{N}$(0, 0.15) |
| SECONDARY SIGNATURES | |
| MODALITY LAYER | |
| connectivity_score | $c_{i}\mathcal{\sim B}\left( 5, 1.2 \right)$ for $i\in\left[ 1, 300 \right]$  $c_{i}\mathcal{\sim B}\left( 1.5, 3 \right)$ for $i\in\left[ 301, 500 \right]$ |
| mod_transition | default |
| nr_noise | 0.02 |
| SUBMODALITY LAYER | |
| submod_transition | cop |
| copula_sm | Frank |
| rho | 0.9 |
| eps_sm | 0.001 |
| prop_sm_up | {0.777; 0.145; 0.078} |
| prop_sm_down | {0.610; 0.301; 0.089} |
| PROBABILITY LAYER | |
| proba_transition | cop |
| copula_prob | Frank |
| theta_prob | 10 |
| nbins_prob | 1e3 |
| qf_vect_up | $\Gamma$(*k*, 0.2) with *k* $\in$ {4.92; 10.40; 12.90} |
| qf_vect_down | $-\Gamma$(*k*, 0.2) with *k* $\in$ {1.67; 6.32; 10.06} |
| qf_vect_nr | $\mathcal{N}$(0, 0.15) |

Table S5: Parameters inspired of an averaged* real data distribution for benchmarking of connectivity scores

| **PARAMETERS** | **VALUES** | |
| --- | --- | --- |
| nb_ent | 19338 | |
| PRIMARY SIGNATURES | | |
| MODALITY LAYER | | |
| p_up | 0.05 | 0.1 |
| p_down | 0.15 | 0.1 |
| SUBMODALITY LAYER | | |
| prop_sm_up | {0.669, 0.246, 0.085} | |
| prop_sm_down | {0.669, 0.246, 0.085} | |
| PERCENTILE LAYER | | |
| qf_vect_up | $\Gamma$(*k*, 0.2) with *k* $\in$ {4.45; 11.13; 13.84} | |
| qf_vect_down | $-\Gamma$(*k*, 0.2) with *k* $\in$ {4.45; 11.13; 13.84} | |
| qf_vect_nr | $\mathcal{N}$(0, 0.15) | |
| SECONDARY SIGNATURES | | |
| MODALITY LAYER | | |
| connectivity_score | $c_{i}\mathcal{\sim B}\left( 5, 1.2 \right)$ for $i\in\left[ 1, 300 \right]$  $c_{i}\mathcal{\sim B}\left( 1.5, 3 \right)$ for $i\in\left[ 301, 500 \right]$ | |
| mod_transition | default | |
| nr_noise | 0.02 | 0.1 |
| SUBMODALITY LAYER | | |
| submod_transition | cop | |
| copula_sm | Frank | |
| rho | 0.9 | |
| eps_sm | 0.001 | |
| prop_sm_up | {0.669, 0.246, 0.085} | |
| prop_sm_down | {0.669, 0.246, 0.085} | |
| PROBABILITY LAYER | | |
| proba_transition | deterministic | |
| qf_vect_up | $\Gamma$(*k*, 0.2) with *k* $\in$ {4.45; 11.13; 13.84} | |
| qf_vect_down | $-\Gamma$(*k*, 0.2) with *k* $\in$ {4.45; 11.13; 13.84} | |
| qf_vect_nr | $\mathcal{N}$(0, 0.15) | |

*In order to obtain a symmetrical distribution, we averaged the complementary modes (absolute value) from up and down modalities. For this matter both modalities need to have the same number of modes. The first mode of this symmetrical distribution was determined by taking the mean value between the average (absolute value) non-deregulated modes and the average of the first up and down modes.

All the modes were estimated following the guidelines outlined in Note S2.

Table S6: Parameters inspired of an averaged* real data distribution for benchmarking of connectivity scores

|  | Kullback-Leiber (KL) divergence | KL divergence ratio relative to biological replicates |
| --- | --- | --- |
| Biological replicates* | 0.1221385 | 1 |
| Simulated VS Real1** | 0.1855657 | 1.52 |
| Real2 VS Real1** | 0.6502182 | 5.32 |

* As the Kullback-Leiber divergence is asymmetrical, we calculated the mean value for the biological replicates extracted from the control samples from NCBI [GEO](https://www.ncbi.nlm.nih.gov/geo/) database accession GSE182024.

** Real1 and Real2 were extracted from NCBI [GEO](https://www.ncbi.nlm.nih.gov/geo/) database accession [GSE185985](https://www.ncbi.nlm.nih.gov/geo/query/acc.cgi?acc=GSE185985) and correspond to treatments with TLN468 and G418, respectively.

**Figure S4 : Average precision score as a function of the number of positively labeled signatures in the evaluation dataset (Top N).** Mean of the average precision scores were calculated over 20 replicates, and the error bars represent the 95% confidence interval. Datasets inspired from averaged real-data distribution (Table S5) except for the deregulated genes proportions and the noise factor.

1. Dataset generated with symmetrical deregulated genes proportions (p_up=0.1; p_down = 0.1) and nr_noise = 0.02
2. Dataset generated with symmetrical deregulated genes proportions (p_up=0.1; p_down = 0.1) and nr_noise = 0.1
3. Dataset generated with asymmetrical deregulated genes proportions (p_up=0.05; p_down = 0.15) and nr_noise = 0.1


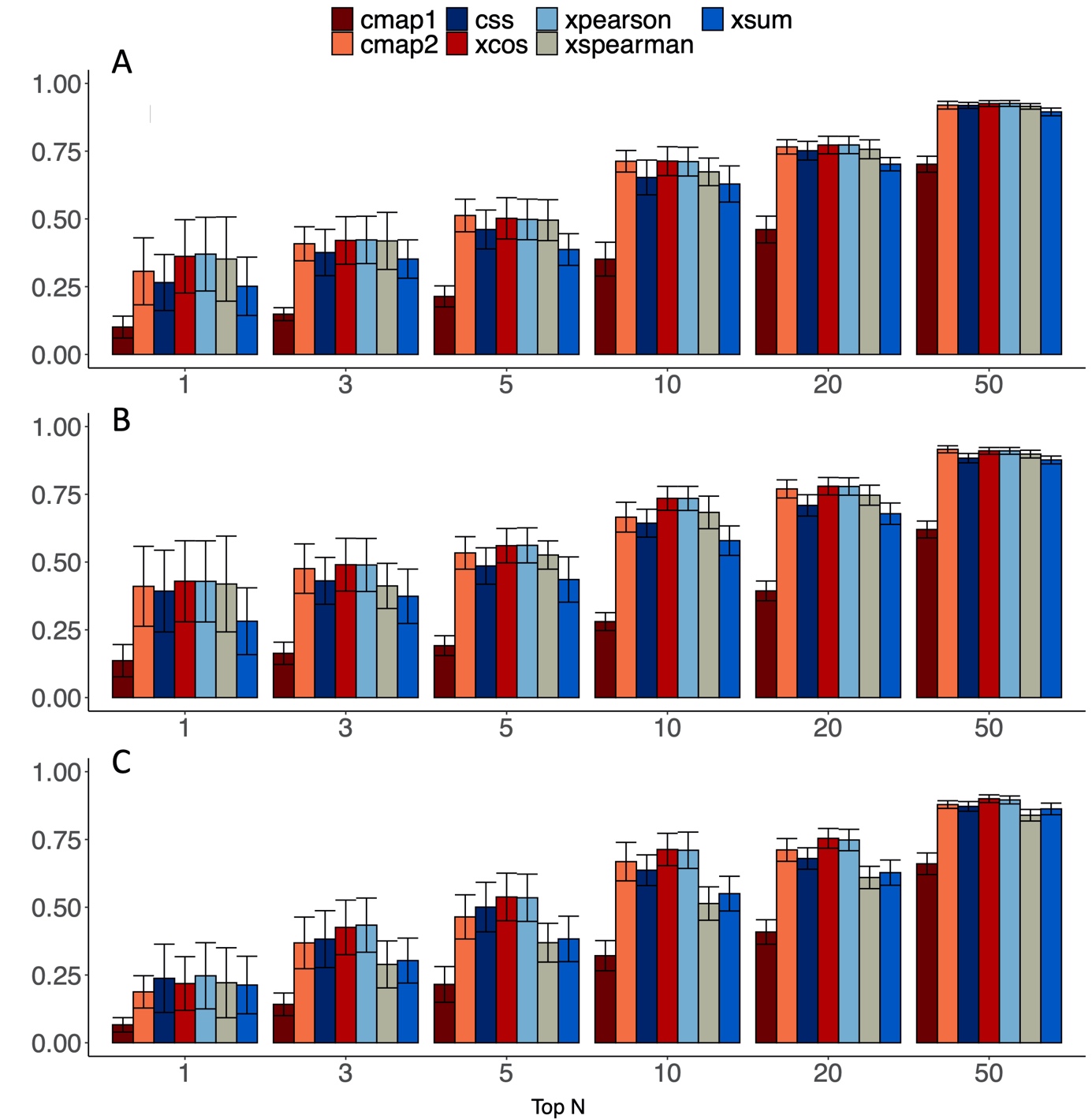


Note S3: Computational resources

The computational cost of the simulation, in terms of memory and calculation time, is really manageable. It varies in function of the number of signatures simulated and the parametrization but here are some references.

Simulation time of one signature (in ms):

|  | **Min** | **Lower Quartile (25%)** | **Mean** | **Median** | **Upper Quartile (75%)** | **Max** | **neval** |
| --- | --- | --- | --- | --- | --- | --- | --- |
| **Primary Signature** | 248.9681 | 261.0145 | 265.403 | 264.2129 | 266.9322 | 307.5775 | 100 |
| **Secondary Signature (independent transition)** | 475.5244 | 493.7425 | 502.9562 | 501.0302 | 507.7964 | 552.1161 | 100 |
| **Secondary Signature (copula transition)** | 24449.12 | 25395.57 | 26174.82 | 26026.44 | 26801.93 | 30029.62 | 100 |

RAM memory use across the simulation of one signature:


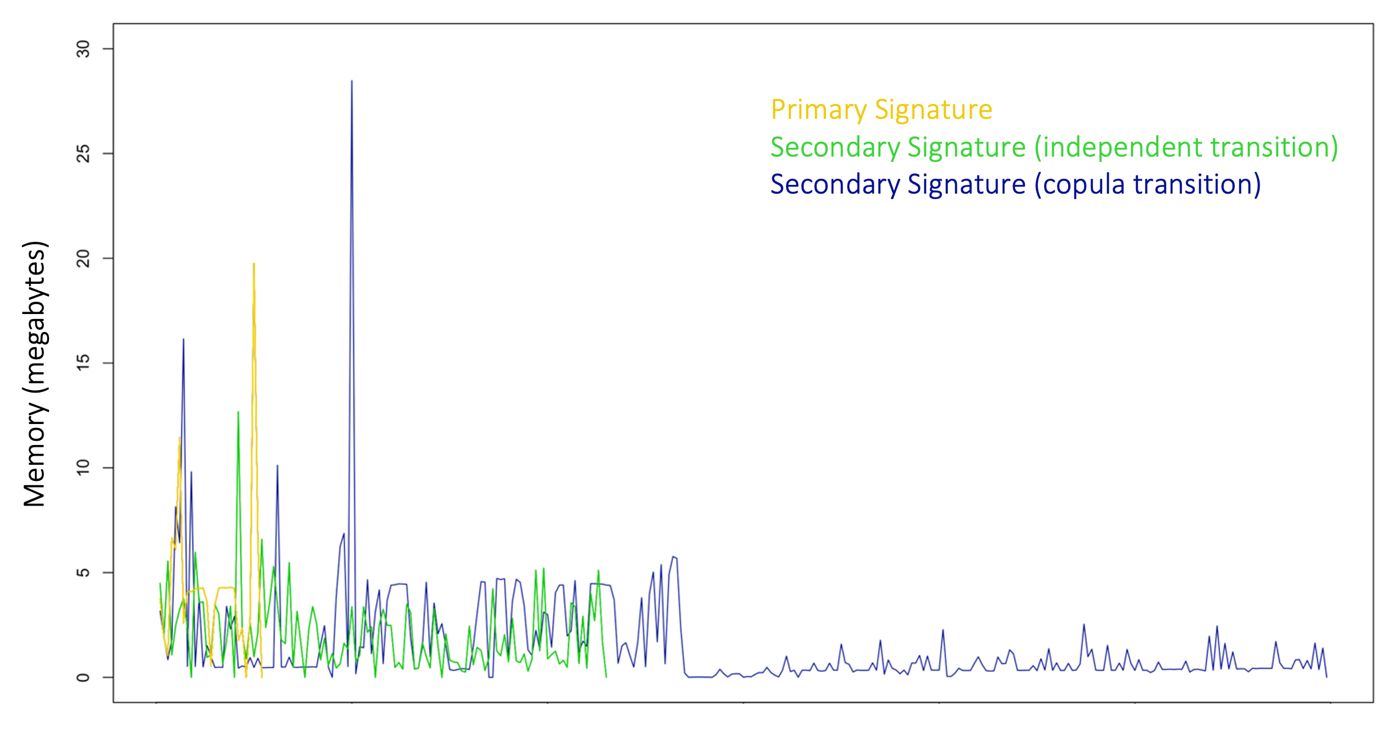


*This metrics were calculated using 7 CPUS, a 2,2 GHz Intel Core i7 four cores processor and a 16Go 1600 MHw DDR3 memory.

Memory usage :

A dataset of 10000 log2 fold-change signatures, each comprising 18856 genes, occupies 1GB of memory saved as a R data frame in RDS format and 1.4GB when it is loaded.

**Figure S5 : Impact of the inference of the control base expression**


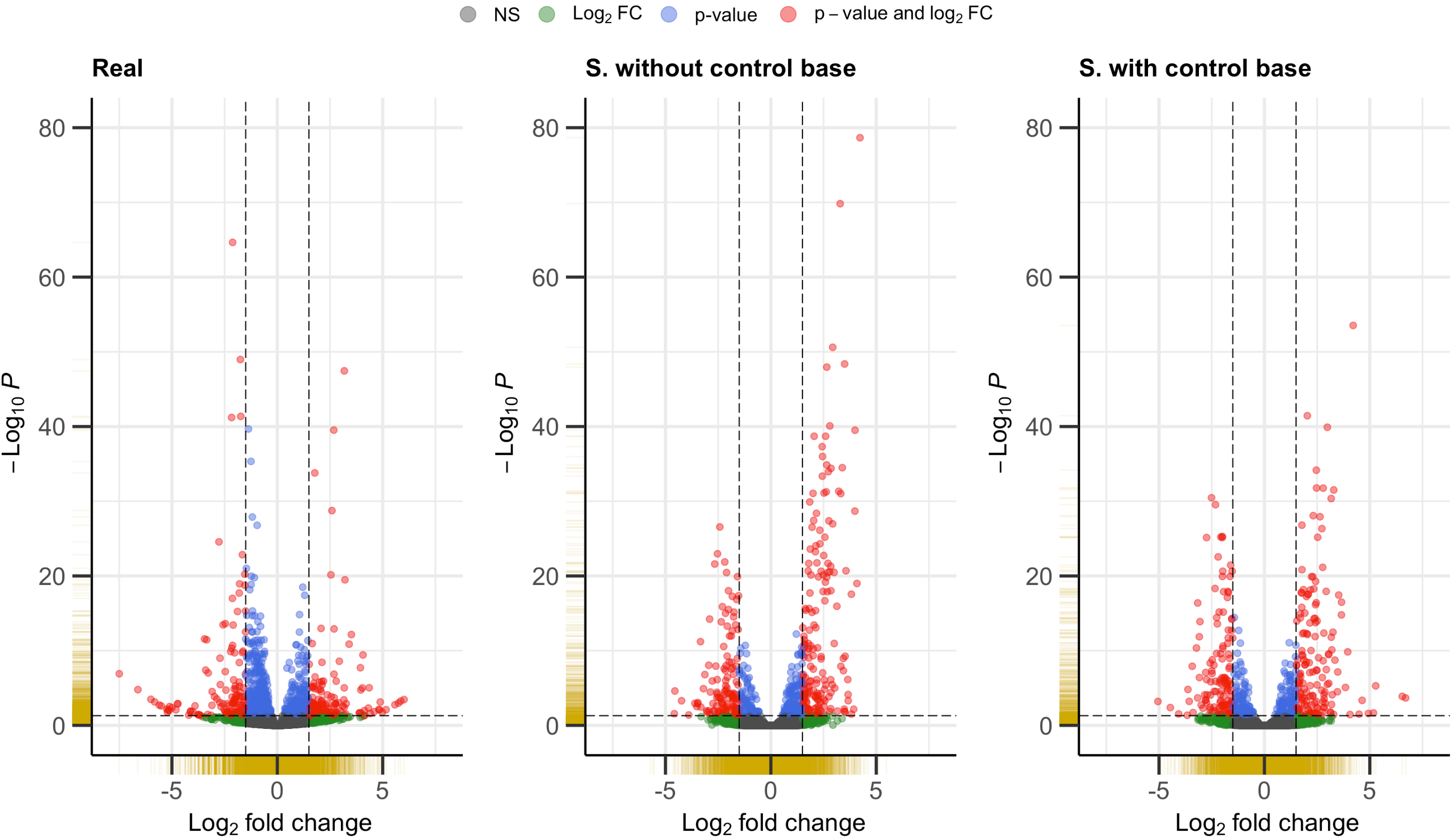


Adjusted p-values were obtained using Benjamini and Hochberg method and filtered at -log_10_P < 80.

1. Michael I Love, Wolfgang Huber, and Simon Anders, “Moderated Estimation of Fold Change and Dispersion for RNA-Seq Data with DESeq2,” 2014, 21, https://doi.org/10.1186/s13059-014-0550-8. [↑](#footnote-ref-2)
2. Byron Hall et al., “LapacesDemon: Complete Environment for Bayesian Inference,” R, 2021, https://web.archive.org/web/20150206004624/http://www.bayesian-inference.com/software. [↑](#footnote-ref-3)
3. Hall et al. [↑](#footnote-ref-4)
